# Consumption of Antioxidant-Rich “Cerrado” Cashew Pseudofruit Affects Hepatic Gene Expression in Obese C57BL/6J High Fat-Fed Mice

**DOI:** 10.3390/foods11172543

**Published:** 2022-08-23

**Authors:** Mariana Buranelo Egea, Gavin Pierce, Si-Hong Park, Sang-In Lee, Fabienne Heger, Neil Shay

**Affiliations:** 1 Goiano Federal Institute, Campus Rio Verde, Rio Verde 75901-970, Brazil; 2 Departament of Food Science and Technology, Oregon State University, Corvallis, OR 97330, USA; 3 Department of Flavor Chemistry, Institute of Food Science and Biotechnology, University of Hohenheim, Schloss Hohenheim 1, 70599 Stuttgart, Germany

**Keywords:** *Anacardium othonianum* Rizz, obesity, functional foods, cashew fruit

## Abstract

The pseudofruit of *A. othonianum* Rizzini, “Cerrado” cashew pulp, has been described as rich in flavonoids, phenolic compounds, and vitamin C. The objective of this work was to evaluate the beneficial health effects seen with the addition of “Cerrado” cashew pulp (CP) to an obesogenic high fat diet provided to C57BL/6J male mice. In week 9, the HF-fed group had a significantly higher baseline glucose concentration than the LF- or HF+CP-fed groups. In RNAseq analysis, 4669 of 5520 genes were found to be differentially expressed. Among the genes most upregulated with the ingestion of the CP compared to HF were *Ph1da1*, *SLc6a9*, *Clec4f*, and *Ica1* which are related to glucose homeostasis; *Mt2* that may be involved steroid biosynthetic process; and *Ciart* which has a role in the regulation of circadian rhythm. Although “Cerrado” CP intake did not cause changes in the food intake or body weight of fed mice with HF diet, carbohydrate metabolism appeared to be improved based on the observed changes in gene expression.

## 1. Introduction

Metabolic syndrome (MetS) is defined as the co-occurrence of three of the following five metabolic risk factors: (i) abdominal obesity, with waist circumference as an index of fat accumulation (visceral adipose deposits); (ii) elevated blood glucose levels associated with Type 2 Diabetes Mellitus (T2DM); (iii) hyperinsulinemia and/or impaired glucose tolerance, which is marked by a decrease in insulin responsiveness in key metabolic tissues such as adipose, muscle, and liver cells; (iv) dyslipidemia, defined as elevated levels of total cholesterol, LDL-C, or triglycerides or low levels of HDL-C, and (v) hypertension, defined as systolic blood pressure (BP) ≥ 135 mmHg or diastolic BP ≥ 85 mmHg. Metabolic syndrome has become a pressing public health issue that now affects an estimated 1.9 billion people worldwide [1,2].

Although these risk factors have been well-established, recent findings demonstrate involvement of the gut microbiome in MetS [3]. Therefore, lifestyle, physical activity, and diet are directly linked to the appearance of dysbiosis, a population imbalance of the host’s intestinal microbiota [4]. Changes in gut microbiome composition and diversity result in alterations in the production of secondary metabolites, interfering with the absorption of important compounds, potentially promoting the development of MetS [5].

Higher dietary diversity consisting of an increased intake of low-energy foods such as non-starchy fruits and vegetables has been linked to a decrease in the development of obesity [6]. Obesity itself is among the major risk factors for a number of chronic diseases, including T2DM, cardiovascular disease (CVD), and cancer [2]. As medical interventions to treat and prevent MetS are currently limited, lifestyle approaches represent the most viable option to prevent the development of this condition. Fruits typically contain dietary fiber and bioactive secondary metabolites, such as polyphenols that may be a useful component of this lifestyle approach. There are many edible fruits which, despite containing a plethora of unique phytochemicals, are currently understudied. These fruits may prove an interesting reservoir of dietary compounds which may favorably impact human health.

Fruits from the Cerrado biome, the second largest biome in Brazil, are recognized as sources of bioactive compounds that promote human health [7]. *Anacardium othanianum* Rizzini is a native fruit from the Cerrado biome that is considered a wild variety of *Anacardium ocidentalle*, the species normally used in the production of Brazilian cashew nuts. The fruit of *A. othanianum* Rizz. is divided into two parts: the true fruit that is the nut and the pseudofruit that is the part that contains the edible pulp and that will be the object of research in this work. *A. othanianum* Rizz. pulp, as well as the juice extracted from this material, has been described as a good source of ascorbic acid, flavonoids [8] such as vitexin and hesperindin, and anthocyanins such as cyanidin, delphinidin, pelargonidin and peonidin [9,10]; all of which endow the fruit with high antioxidant activity. Although the pulp is consumed regionally as a fresh fruit and juice, its nutritional composition suggests that the food industry may be interested in commercializing this crop for its potential human health benefits, as suggested by one of our group’s pilot studies using healthy women [11]. In this work we hypothesize that the ingestion of 2-servings/day of “Cerrado” cashew pulp (CP) will promote metabolic health when consumed with a Western-style diet fed to C57BL/6J male mice.

## 2. Materials and Methods

### 2.1. Vegetal Material

The plant materials were collected in a portion of the “Cerrado” region (16°06′12.7″ S, 051°15 55.8″ W, altitude 391 to 16°08′13.7″ S, 051°17′36.8″ W, altitude 580 m) in Montes Claros de Goiás (Goias, Brazil) in September and October of 2018. The collected plant matter was identified and an exsiccate was deposited in the herbarium as previously described by Oliveira et al. [8]. The project has been officially registered under code A9DF35D (SISGEN, Brazil) to have access to the genetic patrimony.

The material was sanitized (6 mL/L of PuryVitta^®^, 0.96% p/p active chlorine for 15 m), and freeze-dried at the Institute Federal Goiano—Campus Rio Verde. The moisture content, lipids using Soxhlet method, proteins (conversion factor = 5.75), ash, carbohydrates by difference, and dietary fiber (K-TDFR-Megazyme enzyme kit) were determined according to official methods [12]. Energy value was calculated using the Atwater conversion factor. The freeze-dried powder was incorporated into a Western-style diet at 2% *w*/*w* by Research Diets (New Brunswick, NJ, USA), to approximate an equivalent 2 servings/day of the fresh cashew pulp by total energy.

### 2.2. Mouse Diet Studies

Thirty-two male C57BL/6J mice (Jackson Laboratory) were randomly divided into two control groups (*n* = 12 each) and one experimental group (*n* = 8 mice) at 6 weeks of age. After a 2-week acclimatization, groups were provided with experimental diets for 10 weeks. These included a low-fat (LF) diet (10% kcal fat by energy) and a HF diet [45% kcal fat (by energy) + 20% kcal sucrose (by energy) + 1% (*w*/*w*) cholesterol] as controls, or a HF diet plus cashew pulp (HF+CP) for 10 weeks. Mice were fed obesogenic diets high in fat, sucrose, and cholesterol (HF). Cashew pulp (~4% of total energy in 2 servings/d) was included such that all diets had the same percentage of macronutrients, but with different fiber and phytochemical content due to the presence of CP. Supplemental Table S1 shows the diet formulations. Mice (4 per cage) were housed for 10 weeks with free access to food and distilled water at a constant temperature (22 ± 2 °C) with a 12-h light/dark cycle. Each week, the body weight of each mouse and diet intake and spillage in each cage were measured. Weekly diet intake per group was calculated using data over 10 weeks. Food efficiency was defined as the average weekly body weight gain/ total energy consumed from each week. At the end of study, mice were fasted for five hours, anesthetized, and euthanized via exsanguination and cervical dislocation. Cardiac puncture was used to obtain blood samples. These samples were incubated on ice for 60 min and centrifuged at 1000× *g* for 15 min at 4 °C, after which serum was collected and stored at −80 °C. Liver, inguinal adipose tissue, and kidney were collected and weighted. A 100 mg piece of liver was cut and fixed in 10% buffered formalin for histological analysis. The remaining tissue was flash frozen on dry ice and stored at −80 °C.

### 2.3. Fasting Glucose

In week nine, mice fasting for four hours were sedated with isoflurane and placed in flat bottomed rodent restraining tubes, tails were disinfected with 70% ethanol wipes and a small incision made, and fasting blood glucose levels were measured using a handheld glucometer (Contour Next EZ, Bayer Healthcare LLC, Mishawaka, IN, USA). The test was performed mid-day in the middle of the light cycle. 

### 2.4. Hepatic Histological and Total Triglycerides Analysis

Liver tissue was fixed in 10% buffered formalin and embedded in paraffin. Two 5-μm thick sections of each liver were transferred to numbered slides, and liver sections were then stained with hematoxylin-eosin (Nationwide Histology, Spokane, WA, USA). Images were acquired using an Olympus IX71 light microscope (Olympus America, Center Valley, PA, USA).

Liver tissue was homogenized (~100 g) in NP40 Substitute Assay Reagent (Cayman Chemicals, Ann Arbor, MI, USA), followed by centrifugation for 10 min at 10,000× *g* at 4 °C. The supernatants were diluted 1:10 in NP40 Substitute Assay Reagent and assayed using a triglyceride colorimetric assay kit (reference 10010303; Cayman Chemicals).

### 2.5. Serum ELISA

MCP-1 levels were measured in 96-well plates using enzyme-linked immunosorbent assay (ELISA) kits per manufacturer’s instructions (MCP-1 ELISA kit, Invitrogen Corporation, Camarillo, CA, USA). Immediately following the completion of an assay, plates were measured on a Luminex 200 instrument using xPONENT^®^ software (version 4.2, Luminex Corp, Austin, TX, USA).

### 2.6. RNA-Sequencing

#### 2.6.1. RNA Extraction, Library Preparation, and Sequencing

At necropsy, liver tissue samples were collected and stored at −20 °C. Liver tissue (~50 mg) was used to extract total liver RNA with Trizol reagent (Invitrogen, Carlsbad, CA, USA) following the standard protocol. A NanoDrop 2000 spectrophotometer (Thermo Scientific, Carlsbad, CA, USA) was used to analyze the quantity and quality of the isolated RNA and samples (*n* = 3) from each group with the highest RIN, λ260/280, and λ260/230 score were used for RNAseq. Sequencing libraries were generated using the NEBNext^®^ Ultra™ RNA Library Prep Kit for Illumina^®^ (NEB, Ipswich, MA, USA) following the manufacturer’s standard protocols. The six constructed mRNA libraries were sequenced on an Illumina HiSeq 2000 (Illumina, San Diego, CA, USA) at Novogene Technology Co., Ltd. (Novogene Gene Technology, Davis, CA, USA).

#### 2.6.2. Differential Expression Analysis

RNA sequencing and analysis was performed as described previously [13]. To identify genes likely to be direct targets of ligand-activated transcription factors, we evaluated mRNAs known to be regulated by AhR (Aryl hydrocarbon Receptor) [14], CAR (Constitutive Androstane Receptor) [14], PPARα (Peroxisome Proliferator Activated Receptor α) [14], PPARγ (Peroxisome Proliferator Activated Receptor γ) [15], Nrf2 (Nuclear factor erythroid-2 related factor 2) [14], PXR (Pregnane X Receptor) [14,16], LXR (Liver X Receptor) [17,18], and FXR (Farnesyl X Receptor) [19] as described previously by our group [13]. Due to the multiple compositional differences in the two control diets, we choose in this report not to detail the differences between LF- and HF-fed mice.

### 2.7. Microbiome Profiling

Stool samples were collected from each cage (housing four mice) and DNA was isolated using QIAamp Fast DNA Stool Mini Kit (Qiagen, Hilden, Germany) according to the manufacturer’s protocol. The library was prepared following the previous report [20,21] and sequenced using an Illumina MiSeq (2 × 250 bp, 500 cycles) at the Center for Qualitative Life Sciences (CQLS) at Oregon State University (Corvallis, OR, USA). Quantitative Insights into Microbial Ecology 2 (QIIME2) open source pipeline was used to analyze microbiome [22].

### 2.8. Statistical Analysis

Mean ± SEM was used to present the data. Data sets was analyzed using one-way ANOVA and post hoc differences were evaluated using a Tukey’s test (significance was defined as *p* ≤ 0.05 and a trend toward significance was defined as 0.05 < *p* ≤ 0.10). All statistical analyses were carried out using GraphPad Prism 6 software (version 6.0, GraphPad Software, San Francisco, CA, USA).

## 3. Results

### 3.1. Proximal Composition of Cashew Pulp (CP)

The moisture, carbohydrate, total dietary fiber, protein, lipid, and ash contents of CP were 87.99 ± 0.74, 10.11 ± 0.83, 5.89 ± 0.61, 1.04 ± 0.05, 0.52 ± 0.06, and 0.33 ± 0.0 g/100 g, per wet weight, respectively. The energy totaled 49.32 ± 2.77 kcal/100 g.

### 3.2. Energy Intake and Weight Gain

**Figure 1:**
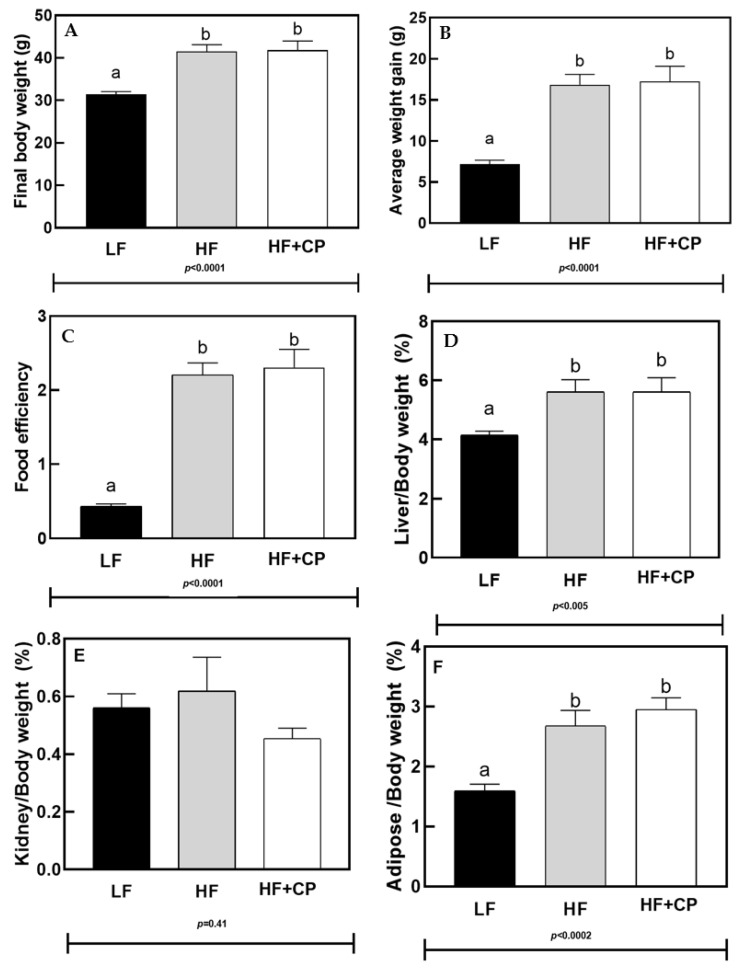
Final body weight (**A**), Average weight gain (**B**), Food efficiency (**C**), Liver weight/body weight (**D**), Kidney weight/ body weight (**E**), and Adipose tissue weight/body weight (**F**) in male C57BL/6J mice fed a low fat (LF) diet, a high fat (HF) diet, or a HF plus cashew pulp (HF+CP) diet for 10 weeks. a, b Groups not sharing the same lowercase letters indicate that one-way ANOVA found significant differences between groups (*p* < 0.05). Average (*n* = 12 for control groups and *n* = 8 for experimental group) ± SEM.

The final body weight and weight gained (Figure 1A,B), as well as the food efficiency, (Figure 1C) did not differ between the HF- and HF+CP-fed groups. Ratios of kidney, liver, and intraperitoneal adipose weights to total body weight are shown in Figure 1D–F, respectively. The HF-diet and HF+CP-diet increased both the liver and adipose to body weight ratios compared to LF-diet mice (*p* = 0.005 and *p* < 0.0002, respectively) while there was no significant difference between the diets for the ratio of kidney/total body weight. 

### 3.3. Biochemical Parameters

**Figure 2:**
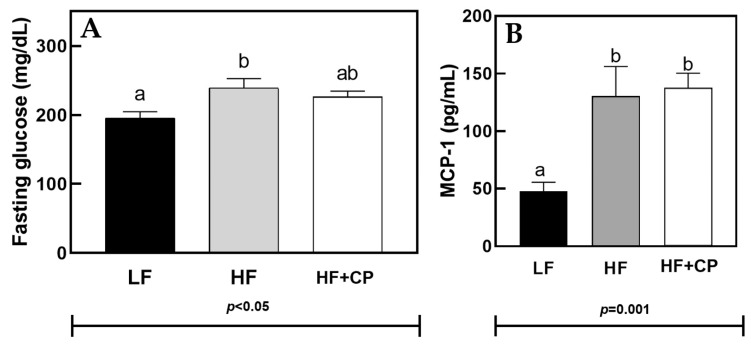
Fasting glucose (**A**) and Serum MCP-1 concentration (**B**) in male C57BL/6J mice fed a LF diet, HF diet, or a HF+CP HF+CP diet for 10 weeks. a, b Groups not sharing the same lowercase letters indicate that one-way ANOVA found significant differences between groups (*p* < 0.05). Average (*n* = 12 for control groups and *n* = 8 for experimental group) ± SEM.

Figure 2 shows the results obtained from the baseline glucose measurements (Figure 2A), taken during week 9. Final week fasting glucose levels were ameliorated in the HF+CP-fed mice to such a degree such that the level became statistically indistinguishable from the LF-fed mice. This statistical difference is distinct from the HF-fed group, which had statistically higher fasting glucose levels compared to the LF group.

The serum MCP-1 concentration was statistically higher in HF-fed and HF+CP-fed groups than the LF-fed group (*p* = 0.001) (Figure 2B).

**Figure 3:**
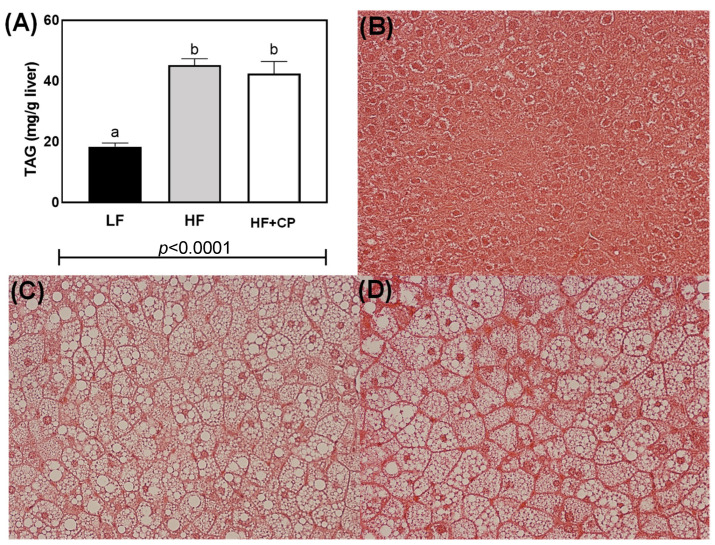
Total triacylglycerol (TAG) assay (**A**) and study hematoxylin-eosin stained liver cross sections of male C57BL/6J mice fed a LF diet (**B**), a HF diet (**C**), or a HF+CP diet (**D**) for 10 weeks. Slides were observed under 400 magnification (40× objective) using an Olympus IX71 light microscope (Olympus America, Center Valley, PA, USA). One-way ANOVA indicated significant differences between diet groups (*p* < 0.05). a, b Groups not sharing the same lowercase letters indicate that one-way ANOVA found significant differences between groups (*p* < 0.05). Average (*n* = 12 for control groups and *n* = 8 for experimental group) ± SEM.

Total triacylglycerol concentration was statistically greater in HF- and HF+CP-fed groups vs. LF-fed mice (*p* < 0.0001) (Figure 3A). Tis is confirmed by visual inspection of corresponding microphotographs of the stained liver tissue (Figure 3B–D).

### 3.4. Gene Expression

Supplementary Figure S1 shows the quality control data from the Transcriptome Analysis Console (TAC) Software for the RNAseq of liver tissue in male C57BL/6J mice fed a LF, HF, or HF+CP diet after 10 weeks. By principal component analysis, axis 1 explained 21.5%, axis 2 explained 14.0%, and axis 3 explained 13.1% of the variance in the original data (A). The quality control of the data was considered good compared with hybridization controls and for target preparation controls (B and C). High separation of positive and negative controls was found in the probe set (D) and box plot (E). 

**Figure 4:**
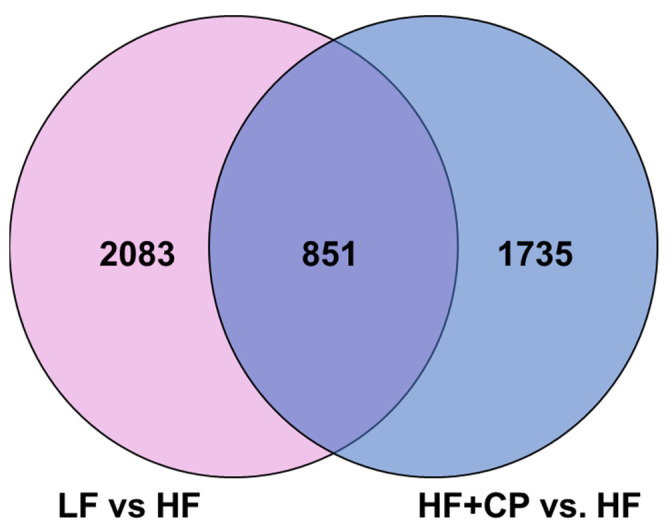
Venn diagram with gene expression of liver of male C57BL/6J mice fed either a LF diet or HF+CP.

A total of 5520 different transcripts were analyzed (Figure 4). 4669 were differentially expressed (*p* < 0.05), with 2083 unique expressed genes for LF- vs. HF-fed groups, 1735 genes unique expressed HF+CP- vs. HF-fed groups, and 851 genes expressed by both LF vs. HF and HF+CP vs. HF.

The 25 canonical pathways most impacted by the HF+CP-group compared to the HF-group are shown in Table 1. For HF+CP vs. HF, it was apparent that many pathways related to immunity, protein regulation, cell signaling, and lipid and glucose metabolism were modulated by the addition of Cerrado cashew pseudofruit to the HF diet.

Lists of the 25 most up- and down-regulated genes for HF+CP-fed group compared to HF-fed group are shown in Table 2. Expression ratios for CP/HF include genes up-regulated with function of cell regulation and organization as *Myc*, *Foxq1*, and *Sdc3*; glucose homeostasis signaling genes such as *Clec4f*, *Wdr46*, *Phlda1*, and *Ica1*; immune response signaling genes such as *Clec4g*, *Fcgr2b*, *H2-Ab1*, and *Fcgr3*; hormone regulation *Usp2* and *Mt2*; circadian rhythm regulation gene as *Ciart*; and transport-related genes including *Slc6a9*. Expression ratios for CP/HF include genes down-regulated with function of lipid homeostasis as *Cyp2b9*, *G0s2*, *Cyp2b10*, *Cyp2b13*, *Fabp2*, *Acly*, *Faap20*, and *Fgfr4*; genes responsible for the immune response as *Golm1* and *Leap2*, activators of proinflammatory cytokines as *Gdf15* and *Atf5*; and cell homeostasis as *Psen2*, *Cyr61*, *Col3a1*, *Sco2*, *Kdm4a*, and *Rnd1*.

### 3.5. Microbiome Profile

**Figure 5:**
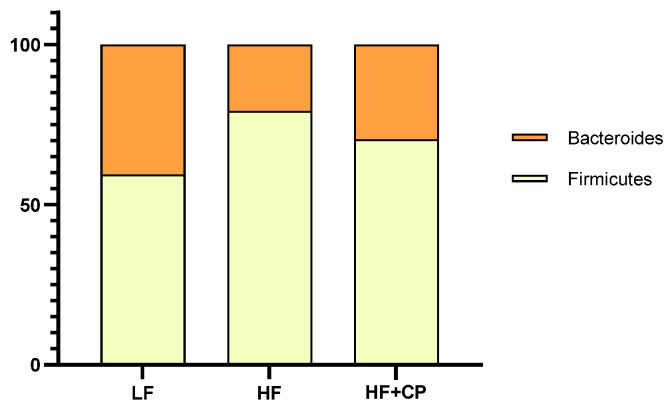
Bacteroids:Firmicutes ratio of microbiome of male C57BL/6J mice fed a low fat (LF) diet (**A**), a high fat (HF) diet (**B**), or a HF plus cashew pseudofruit (HF+CP) diet.

PCA was used to analyze the relative variation in the microbiome of each group profiled from the stool sample. The relative abundance of phyla and 10 most prolific bacteria are listed in Table 3. At the phylum level, while *Actinobacteria* abundance was significantly higher in HF- vs. LF-fed mice, *Actinobacteria* abundance in the HF+CP-fed group was statistically indistinguishable from the LF-fed mice (*p* = 0.001) (Figure 5). Conversely, Bacteroidetes was elevated in LF- compared with HF- and HF+CP-fed groups, and all the fed groups showed significantly different percentages between them. *Firmicutes*, *Proteobacteria*, and *Verrucomicrobia* were not different between LF-, HF-, and HF+CP-fed groups.

At genus level, there were no differences observed in the taxa *Akkermansia*, *Clostridiales*, *Bacteroides*, *Ruminococcaceae*, and *Sutterella* between LF-, HF-, and HF+CP-fed groups. The LF-fed control group showed significantly more abundance of bacteria from the family S24-7 than all other groups (*p* < 0.0001), while HF- and HF+CP-fed groups were indistinguishable statistically. The *Coriobacteriaceae* and *Lactococcus* abundance were significantly higher in HF- compared with LF- and HF+CP -fed groups, while LF- and HF+CP-fed groups were statistically indistinguishable. Finally, *Oscillospira* abundance was significantly higher in HF+CP-fed group, while LF- and HF-fed groups were statistically indistinguishable.

## 4. Discussion

Feeding a Western-style diet containing elevated levels of total fat, cholesterol, and sucrose to the mice significantly increased all measured physiological and biochemical parameters such as final body weight, weight gain, food efficiency, tissue weights, fasting glucose, glucose and MCP-1 concentration in the serum, and triglycerides in the liver. The group of mice fed cashew pulp showed similar fasting glucose compared with LF-fed mice, which is remarkable considering the CP-fed group was consuming elevated levels of total fat, cholesterol, and sugar in comparison to the low-fat and low-sugar LF diet. This is an important finding that has not been previously reported. In future studies, serum insulin concentrations should be measured to verify the changes in blood glucose we observed in this study. In the present study, insulin testing was inconclusive, in part due to a lack of serum to successfully complete an ELISA.

Regarding the regulation of gene expression, it is known that LXR can influence transcription of *Abca1*, *ApoE/CII*, *Pltp*, *Abcg1*, and other *Abcg* family members; if indeed upregulated, these changes should increase cholesterol efflux [23]. Although the literature states that the increased transcription of *Abcg5* and *Abcg8* genes may lead to a 50% reduction in cholesterol absorption [24], this was not observed in the present work. This effect is usually associated with increased fiber in the diet [25] and in this study the fiber content was relatively similar between the HF- and HF+CP-fed groups. Cashew pulp showed 5.89 g/100 g of dietary fiber, resulting in 9.42 g in 2 serving portions included in the mice diet. Considering the Recommended Dietary Allowances [26] for men and women (38 and 25 g/day, respectively), two servings of cashew pulp could provide ~25 and ~38% of the RDA of dietary fiber for men and women, respectively. However, in the case of “Cerrado” cashew it seems that the cholesterol-lowering effect may be associated with the bioactive compounds of the fruit since previously our group showed a tendency to decrease total cholesterol and triglycerides in a pilot study with women [11]. The change in serum glucose when mice were fed “Cerrado” cashew may be related to activation of LXR (Supplementary Table S2) that plays an essential role in glucose homeostasis [27]. 

It is possible that consumption of the HF diet activated hepatic stellate cells (HSCs). Genes upregulated in mouse liver with the HF+CP-diet demonstrated a strong tendency to activate immunogenic function by antigen-dependent and independent mechanisms [28,29]. Some genes upregulated with the addition of “Cerrado” cashew pulp have demonstrated functions related to this mechanism such as *Clec4g*, a ligand for CD44, which can inhibit T cell activation and proliferation or induce apoptosis, contributing to the prevention of immune-mediated liver damage [28]. Moreover, the expression of *Kdr*, a vascular endothelial growth factor, can induce proliferation of HSCs required for hepatic tissue and fibrosis resolution [29]. 

Diurnal rhythms and locomotor activity can be impacted in obesity rodent models [30]. The upregulation of *Ciart,* which participates in circadian rhythm, in the present work demonstrated that the addition of cashew in HF diet may be influencing the regulation of circadian rhythms. The circadian clock plays an important role in controlling body weight due to expression and secretion of hormones such as melatonin [30]. However, changes in body weight were not seen in this work, perhaps due to the duration of the study.

The dietary administration of cashew changed the intestinal microbiota populations from a predominance of *Firmicutes*, *Actinobacteria*, and *Verrucomicrobia* to a predominance of *Firmicutes*, *Verrucomicrobia*, and *Bacteroidetes* for the high fat diet group. Although low relative frequencies of *Bacteroidetes* have been attributed to high fat diet [31], the cashew fed group showed ~19% vs. ~13 % for this HF-fed group, which appears to be an improvement caused by ingestion of cashew pulp. *Bacteroides* often produce acetate and propionate which have been demonstrated to exert protective effects against diet-induced obesity, improve glucose tolerance, and act as signaling molecules that activate pathways such as LXR signaling [32,33]. We hypothesize that the change in the profile of the gut microbiota correlates with the apparent activation of LXR demonstrated in the significantly regulated hepatic mRNA list provided in Supplementary Table S2.

*Bacteroidales S24-7* had significantly higher presence in the LF-fed control group, suggesting that these bacteria may be helpful for sustaining a healthy metabolism as described in another work from our group [34]. Although there is no significant difference between the HF- and HF+CP-fed groups, the relative frequency of *S24-7* was higher when cashew was ingested. This group of bacteria has a negative correlation with intestinal inflammation [35], which may be involved in the increase in target transcription of immune-related genes found in the present work.

*Akkermansia*, a genus in the *Verrucomicrobia* phylum, showed a tendency to increase (*p* = 0.25) and was here demonstrated to be the most populous when cashew was added to the HF-diet of mice for 10 wks. *Akkermansia muciniphila* is a species of the human and mouse intestinal microbiome, and the most abundant member of the genus. This species has been associated with increased mucus layer thickness, and improved glucose homeostasis and cardiometabolic parameters in models of obesity [36,37]. *Oscillospira* showed a relative frequency almost twice as high in the HF+CP- than the HF-fed group. Although the abundance of *Oscillospira* has been linked to leanness [38], in the present work no difference in weight gain and final body weight was found (Figure 1).

In obese male mice, we hypothesize that the flavonoids and anthocyanins in the “Cerrado” cashew pseduofruit, are beneficially impacting metabolism, improving fasting blood glucose and changing hepatic target transcription, especially related to FA metabolism, the immune system, and glucose homeostasis. Flavonoids such as kaempferol and epicatechin [8] or anthocyanins such as cyanidin and delphinidin [9] that are present in the “Cerrado” cashew, have already been reported in the literature for having an important role as anti-obesity and anti-diabetic potential compounds [39,40,41,42] in obese C57BL/6J high fat-fed mice. However, to confirm this hypothesis, further studies are needed with increased numbers of animals and the study of female animals as well.

## Figures and Tables

**Table 1:** Canonical pathways enriched for induced the down-regulated and up-regulated genes of liver from male C57BL/6J mice fed HF+CP.

	Pathway	#Total	#Up	#Down	Significance	*p*-Value
1	Complement Activation, Classical Pathway (part of Immune system)	8	7	1	3.46	0.000
2	Cytoplasmic Ribosomal Proteins	21	4	17	3.45	0.000
3	G Protein-coupled Receptor (GPCR)	11	3	8	2.77	0.002
4	Complement and Coagulation Cascades	16	14	2	2.64	0.002
5	MAPK Cascade	9	5	4	2.3	0.005
6	Odorant GPCR	14	8	6	2.17	0.007
7	Non-odorant GPCR	18	8	10	2.13	0.007
8	MAPK signaling pathway	29	18	11	1.74	0.018
9	Metapathway biotransformation	8	6	2	1.72	0.019
10	Nuclear receptors in lipid metabolism and toxicity	8	7	1	1.69	0.021
11	TYROBP Causal Network	13	12	1	1.65	0.022
12	MAPK signaling pathway	30	21	9	1.64	0.023
13	Microglia Pathogen Phagocytosis Pathway	10	10	0	1.61	0.025
14	Robo4 and VEGF Signaling Pathways Crosstalk	3	3	0	1.6	0.025
15	miRNAs involved in DNA damage response	1	1	0	1.59	0.026
16	Glycolysis and Gluconeogenesis	1	1	0	1.58	0.026
17	Novel Jun-Dmp1 Pathway	7	5	2	1.55	0.028
18	Mitochondrial LC-Fatty Acid Beta-Oxidation	5	3	2	1.48	0.033
19	PPAR signaling pathway	16	11	5	1.41	0.039
20	Fatty Acid Beta Oxidation (streamlined)	8	5	3	1.32	0.047
21	Toll-like receptor signaling pathway	18	13	5	1.23	0.058
22	Steroid Biosynthesis	4	4	0	1.23	0.059
23	Focal Adhesion	32	22	10	1.19	0.064
24	GPCRs, Class A Rhodopsin-like	18	9	9	1.19	0.065
25	Eicosanoid Synthesis	5	4	1	1.18	0.066

**Table 2:** Top 25 upregulated and downregulated genes of liver from male C57BL/6J mice fed HF+CP after 10 weeks. FC: Fold Change.

No	Upregulated Genes				Downregulated Genes			
	FC	*p*-Value	Gene	Function	FC	*p*-Value	Gene	Function
1	12.00	4.82 × 10^−2^	*Myc*	cell cycle progression, apoptosis, and cellular transformation	−12.98	1.50 × 10^−3^	*Cyp2b9*	synthesis of cholesterol, steroids and other lipids
2	7.87	3.91 × 10^−2^	*Foxq1*	cell cycle regulation	−4.46	4.15 × 10^−2^	*Gdf15*	TGF-beta ligant
3	5.79	4.16 × 10^−2^	*Usp2*	TNF signaling (REACTOME) and Regulation of TP53 Activity	−3.58	2.80 × 10^−3^	*Spp1*	the protein encoded by this gene is involved in the attachment of osteoclasts to the mineralized bone matrix
4	5.66	3.79 × 10^−2^	*Phlda1*	anti-apoptotic effects of insulin-like growth factor-1	−3.19	3.00 × 10^−4^	*G0s2*	regulation of lipid metabolism
5	5.29	6.50 × 10^−3^	*Wee1*	involved in DNA replication	−2.72	6.80 × 10^−3^	*Cyp2b10*	steroid, fatty acid, and xenobiotic compounds oxidation
6	4.24	3.51 × 10^−7^	*Ehd3*	megakaryocyte development and platelet production and response to elevated platelet cytosolic Ca2+	−2.59	1.20 × 10^−3^	*Leap2*	xenobiotic metabolism
7	4.12	5.54 × 10^−5^	*Dnase1l3*	breakdown of DNA during apoptosis	−2.51	3.90 × 10^−3^	*Psen2*	intracellular signaling
8	4.01	6.13 × 10^−6^	*Clec4g*	receptor with an affinity for mannose and fucose	−2.51	1.01 × 10^−7^	*Ucn*	activation of cAMP-Dependent PKA (glucose homeostasis) pathways
9	3.95	2.56 × 10^−5^	*Kdr*	vascular endothelial growth factor (VEGF)	−2.49	1.59 × 10^−2^	*Cyr61*	cell grown and differentiation, and extracellular matrix formation.
10	3.57	4.00 × 10^−4^	*Fcgr2b*	involved in the phagocytosis of immune complexes and in the regulation of antibody production by B-cells	−2.47	9.30 × 10^−3^	*Cyp2b13*	arachidonic acid epoxygenase activity
11	3.49	2.00 × 10^−4^	*Oit3*	downregulation in hepatocarcinom	−2.3	2.66 × 10^−2^	*Pde9a*	cAMP and cGMP regulation
12	3.34	2.80 × 10^−3^	*Por*	synthesis of bile acids and bile salts and cytochrome P450	−2.29	4.72 × 10^−2^	*Col3a1*	extracellular matrix
13	3.34	2.06 × 10^−2^	*Mt2*	steroid biosynthetic process	−2.21	1.48 × 10^−2^	*Sco2*	cell growth
14	3.29	2.29 × 10^−2^	*Ciart*	circadian rhythm	−2.18	1.71 × 10^−2^	*Golm1*	involved with liver disease and autoimmune hepatitis
15	3.14	3.04 × 10^−2^	*Tsku*	among its related pathways are ectoderm differentiation	−2.18	3.00 × 10^−4^	*Rab1b*	cell transportation
16	3.05	6.80 × 10^−3^	*H2-Ab1*	adaptive immunity	−2.09	2.10 × 10^−3^	*Tbca*	hormone regulation
17	3.04	1.90 × 10^−3^	*Slc6a9*	transportation of molecules and NRF2 pathway	−2.02	2.30 × 10^−3^	*Fabp2*	lipid homeostasis
18	3	1.00 × 10^−4^	*Fcgr3*	immune response	−2.01	2.49 × 10^−2^	*Acly*	acetyl-CoA synthesis
19	2.96	2.00 × 10^−3^	*Eng*	TGF-beta component	−1.99	2.57 × 10^−2^	*Gldc*	glucose homeostasis
20	2.96	1.00 × 10^−3^	*Sdc3*	organization and signaling (sugar-dependent mechanism) cellular	−1.98	1.10 × 10^−3^	*Atf5*	IL-2 Pathway/Imune sistem
21	2.86	1.40 × 10^−3^	*Clec4f*	receptor with an affinity for galactose and fucose	−1.96	2.00 × 10^−4^	*Prelid2*	phosphatidic acid transfer activity
22	2.82	3.85 × 10^−5^	*Wdr46*	galactose and carbohydrate binding	−1.95	6.70 × 10^−3^	*Kdm4a*	nuclear protein
23	2.74	2.00 × 10^−3^	*Rasgrp3*	activates the oncogenes HRAS and RAP1A.	−1.93	2.27 × 10^−2^	*Rnd1*	cell regulation
24	2.73	3.00 × 10^−3^	*Ica1*	autoantigen in T2DM (or IDDM)	−1.92	3.40 × 10^−3^	*Faap20*	fatty acid synthesis
25	2.7	9.00 × 10^−4^	*Wfdc17*	activated macrophage/microglia WAP domain protein	−1.92	4.31 × 10^−2^	*Fgfr4*	lipid homeostasis

**Table 3:** Relative abundance of selected bacteria in the cecal microbiome in male C57BL/6J mice fed HF+CP after 10 weeks. a, b Groups not sharing the same lowercase letters indicate that one-way ANOVA found significant differences between groups (*p* < 0.05).

Phyla	LF	HF	HF+CP	*p*-Value
p__Actinobacteria	6.45 ± 0.64 ^b^	16.59 ± 0.43 ^a^	7.02 ± 0.60 ^b^	0.001
p__Bacteroidetes	29.18 ± 0.28 ^a^	13.76 ± 0.66 ^c^	19.41 ± 0.47 ^b^	0.0001
p__Firmicutes	44.26 ± 4.19	52.75 ± 0.66	46.13 ± 0.43	0.46
p__Proteobacteria	1.77 ± 0.38	1.68 ± 0.03	2.20 ± 0.03	0.73
p__Verrucomicrobia	18.34 ± 2.99	15.22 ± 0.57	25.23 ± 0.33	0.25
*Genera*				
g__Akkermansia	18.31 ± 2.97	15.22 ± 0.57	25.23 ± 0.49	0.25
o__Clostridiales	16.90 ± 6.04	11.98 ± 2.17	20.53 ± 0.27	0.15
g__Bacteroides	9.66 ± 0.49	8.22 ± 0.45	11.99 ± 1.06	0.11
o__Bacteroidales;S24-7	19.48 ± 0.24 ^a^	5.54 ± 0.25 ^b^	7.42 ± 0.35 ^b^	<0.0001
f__Coriobacteriaceae	6.38 ± 0.64 ^b^	16.45 ± 0.43 ^a^	6.86 ± 0.87 ^b^	0.001
g__Oscillospira	4.08 ± 0.26 ^b^	4.63 ± 0.24 ^b^	7.18 ± 0.61 ^a^	0.025
g__Lactococcus	4.01 ± 0.41 ^b^	6.84 ± 0.27 ^a^	2.54 ± 0.08 ^b^	0.008
f__Ruminococcaceae	1.91 ± 0.14	3.26 ± 0.28	1.93 ± 0.36	0.10
g__Sutterella	1.77 ± 0.38	1.68 ± 0.03	2.20 ± 0.04	0.73
Others	17.51 ± 4.08	26.18 ± 0.74	14.11 ± 0.16	0.28

## Supplementary Materials

The following supporting information can be downloaded at: https://www.mdpi.com/article/10.3390/foods11172543/s1, Figure S1: Quality control (QC) from Transcriptome Analysis Console (TAC) Software for the RNA sequencing of liver tissue in male C57BL/6J mice fed a low fat (LF) diet, a high fat (HF) diet, or a HF plus cashew pulp (HF+CP) diet for 10 weeks, Table S1: Compositions of Experimental Diets and Serving Information, and Table S2: Most significantly regulated hepatic mRNAs and associated ligand activated transcription factors from male C57BL/6J mice fed high fat (HF) plus cashew pseudofruit (HF+CP) vs. HF diet for 10 weeks
