# Supplementary material for: Consumption of Antioxidant-Rich “Cerrado” Cashew Pseudofruit Affects Hepatic Gene Expression in Obese C57BL/6J High Fat-Fed Mice
Source: Foods. 2022 Aug 23;11(17):2543. doi: 10.3390/foods11172543 (PMC9455023; doi:10.3390/foods11172543)

Supplementary Figure 1. Quality control (QC) from Transcriptome Analysis Console (TAC) Software for the RNA sequencing of liver tissue in male C57BL/6J mice fed either a low fat (LF) diet, a high fat (HF) diet, and HF plus cashew pulp (HF+CP) after 10 weeks

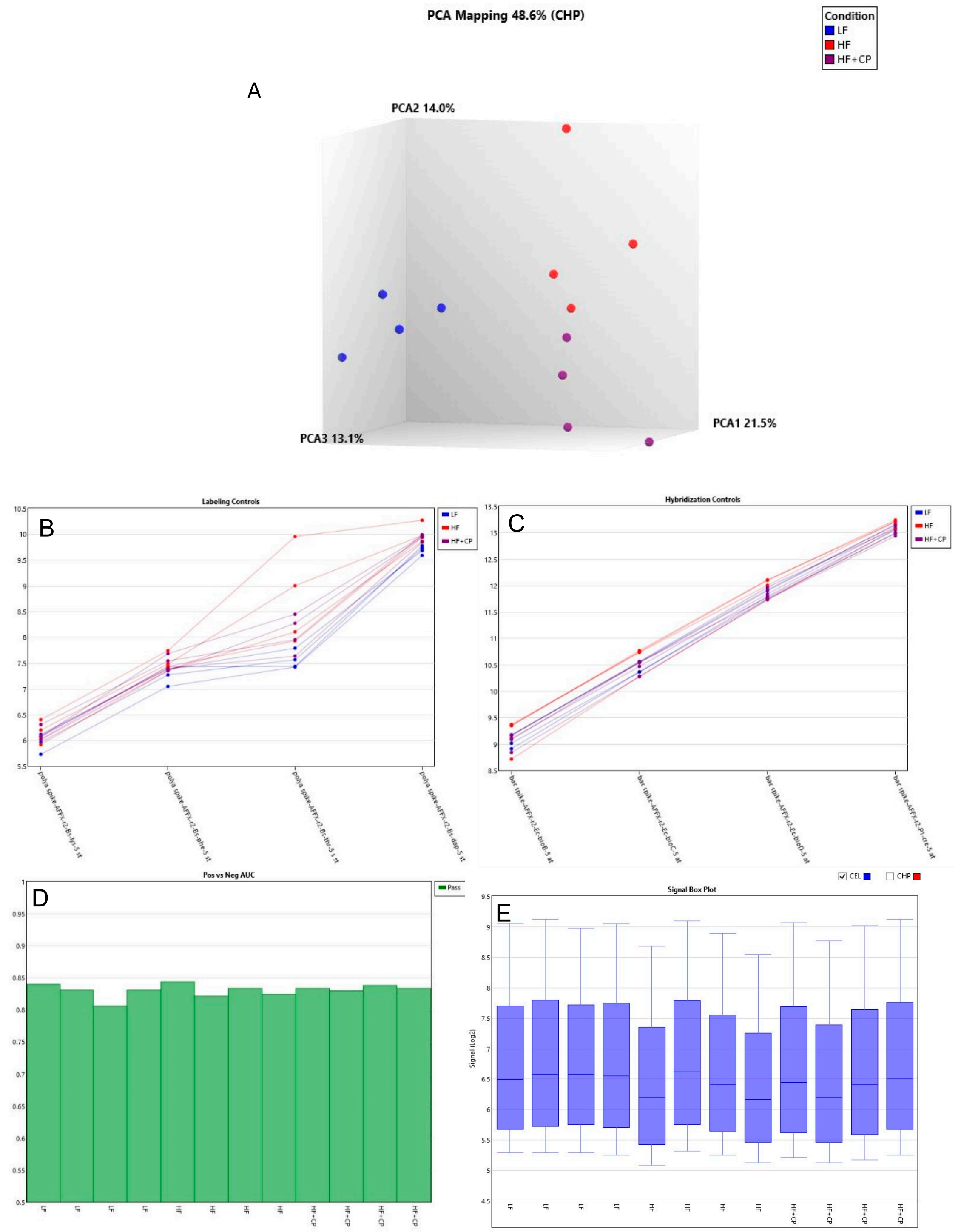

Supplement: Supplementary file 1 [file foods-11-02543-s001.zip › Figure S1.pdf]
