# Supplementary material for: Consumption of Antioxidant-Rich “Cerrado” Cashew Pseudofruit Affects Hepatic Gene Expression in Obese C57BL/6J High Fat-Fed Mice
Source: Foods. 2022 Aug 23;11(17):2543. doi: 10.3390/foods11172543 (PMC9455023; doi:10.3390/foods11172543)
Supplement: Supplementary file 1 [file foods-11-02543-s001.zip › Table S1.pdf]

Table 1 S1. Compositions of Experimental Diets and Serving Information

**Product #**	**HF**	**HF+CP**
	45 kcal% Fat with	*2% Cashew Pulp*
	1% Chol Control	
**Ingredient**	**gm**	**gm**
Casein	200	198.3
L-Cystine	3	3
Corn Starch	72.8	65.5
Maltodextrin 10	100	100
Sucrose	172.8	165.5
Cellulose, BW200	50	49.5
Soybean Oil	25	25
Lard	177.5	176.8
Mineral Mix S10026	10	10
DiCalcium Phosphate	13	13
Calcium Carbonate	5.5	5.5
Potassium Citrate, 1 H_2O	16.5	16.5
Vitamin Mix V10001	10	10
Choline Bitartrate	2	2
Cholesterol	8.5	8.5
Cashew Pulp, Dried	0	17.3
FD&C Yellow Dye #5	0	0.025
FD&C Blue Dye #1	0	0.025
FD&C Red Dye #40	0	0
**Total**	**866.6**	**866.45**
**Total (Dry Basis)**	**866.6**	**866.45**
**g**		
Protein	177.0	177.0
Carbohydrate	355.6	355.6
Sugar	182.8	182.8
Starch	172.8	172.8
Fat	204.9	204.9
Fiber	50.0	50.0

**g%**		
Protein	20.4	20.4
Carbohydrate	41.0	41.0
Fat	23.6	23.7
Fiber	5.8	5.8
Polyphenol #25	0.0	0.0
Polyphenol #80	0.0	0.0
S-Methylmethionine	0.0	0.00
Cashew Pulp, Dried	0.0	2.0
**kcal**		
Protein	*708*	*708*
Carbohydrate	*1422*	*1422*
Fat	*1844*	*1844*
Total	*3975*	*3975*
**kcal%**		
Protein	*18*	*18*
Carbohydrate	*36*	*36*
Fat	*46*	*46*
kcal / gm	**4.59**	**4.59**
