# Supplementary material for: Consumption of Antioxidant-Rich “Cerrado” Cashew Pseudofruit Affects Hepatic Gene Expression in Obese C57BL/6J High Fat-Fed Mice
Source: Foods. 2022 Aug 23;11(17):2543. doi: 10.3390/foods11172543 (PMC9455023; doi:10.3390/foods11172543)
Supplement: Supplementary file 1 [file foods-11-02543-s001.zip › Table S2.pdf]

Table 2 S2. Most significantly regulated hepatic mRNAs and associated ligand activated transcription factors from male C57BL/6J mice fed high fat (HF) plus cashew pseudofruit (HF+CP) vs. HF diet for 10 weeks

	FoldChange	pvalue	GeneName	AhR	CAR	FRX	LXR	PPAR	Nrf2
1	1.35	1.71E-02	*Abca1*				X		
2	1.22	8.47E-02	*Abcc4*	X	X			X	X
3	1.40	1.41E-02	*Abcg1*				X		
4	1.99	8.16E-05	*Abcg5*				X	X	
5	1.20	1.52E-02	*Abcg8*				X		
6	1.39	5.40E-03	*Aldh9a1*					X	
7	2.22	3.60E-03	*Cyp7a1*			X	X		
8	1.30	6.91E-02	*Nr1h3*				X		
9	2.19	3.00E-04	*Papss2*		X			X	
10	1.84	9.10E-03	*Pltp*				X		
11	1.34	3.46E-02	*Slc7a15*			X			
12	2.06	4.50E-03	*Sult5a1*	X	X				
